# Health and human rights in eastern Myanmar prior to political transition: a population-based assessment using multistaged household cluster sampling

**DOI:** 10.1186/1472-698X-14-15

**Published:** 2014-05-05

**Authors:** Parveen K Parmar, Jade Benjamin-Chung, Linda S Smith, Saw Nay Htoo, Sai Laeng, Aye Lwin, Mahn Mahn, Cynthia Maung, Daniel Reh, Eh Kalu Shwe Oo, Thomas Lee, Adam K Richards

**Affiliations:** 1 Department of Emergency Medicine, Brigham and Women’s Hospital, Harvard Medical School, 75 Francis St, Boston, MA 02115, USA; 2 Division of Epidemiology, UC Berkeley School of Public Health, 101 Haviland Hall, Berkeley, CA 94720-7358, USA; 3 Community Partners International, 2560 Ninth St., Suite 315b, Berkeley, CA 94710, USA; 4 Burma Medical Association, PO Box 156, Mae Sot, Tak, Thailand; 5 Shan State Development Foundation, Chiang Mai, Thailand; 6 Back Pack Health Worker Team, Mae Sot, Tak, Thailand; 7 Mae Tao Clinic, Mae Sot, Tak, Thailand; 8 Karenni Mobile Health Committee, Mae Hong Son, Thailand; 9 Karen Department of Health and Welfare, PO box 189, Mae Sot, Tak 63110, Thailand; 10 Adjunct Professor of Medicine, University of California, Los Angeles, CA, USA; 11 Division of General Internal Medicine and Health Services Research, University of California, Los Angeles, CA 90024, USA

**Keywords:** Burma, Myanmar, Human Rights, Health, Mortality, Malaria

## Abstract

**Background:**

Myanmar/Burma has received increased development and humanitarian assistance since the election in November 2010. Monitoring the impact of foreign assistance and economic development on health and human rights requires knowledge of pre-election conditions.

**Methods:**

From October 2008-January 2009, community-based organizations conducted household surveys using three-stage cluster sampling in Shan, Kayin, Bago, Kayah, Mon and Tanintharyi areas of Myanmar. Data was collected from 5,592 heads of household on household demographics, reproductive health, diarrhea, births, deaths, malaria, and acute malnutrition of children 6–59 months and women aged 15–49 years. A human rights focused survey module evaluated human rights violations (HRVs) experienced by household members during the previous year.

**Results:**

Estimated infant and under-five rates were 77 (95% CI 56 to 98) and 139 (95% CI 107 to 171) deaths per 1,000 live births; and the crude mortality rate was 13 (95% CI 11 to 15) deaths per thousand persons. The leading respondent-reported cause of death was malaria, followed by acute respiratory infection and diarrhea, causing 21.2% (95% CI 16.5 to 25.8), 16.6% (95% CI 11.8 to 21.4), and 12.3% (95% CI 8.7 to 15.8), respectively. Over a third of households suffered at least one human rights violation in the preceding year (36.2%; 30.7 to 41.7). Household exposure to forced labor increased risk of death among infants (rate ratio (RR) = 2.2; 95% CI 1.1 to 4.4) and children under five (RR = 2.1; 95% CI 1.3 to 3.6). The proportion of children suffering from moderate to severe acute malnutrition was higher among households that were displaced (prevalence ratio (PR) = 3.3; 95% CI 1.9 to 5.6).

**Conclusions:**

Prior to the 2010 election, populations of eastern Myanmar experienced high rates of disease and death and high rates of HRVs. These population-based data provide a baseline that can be used to monitor national and international efforts to improve the health and human rights situation in the region.

## Background

Ruled by a military junta for decades, Burma/Myanmar (hereafter referred to as Myanmar) held elections in November 2010, and the nominally civilian parliamentary government that took power in March 2011 has overseen a dramatic increase in humanitarian assistance and business investment. Accurate information related to health and human rights conditions under the old governance structure will allow the current government and the international community to monitor the health impact of these policy changes, and to track the progressive realization of the right to health.

National health statistics reported by UNICEF suggest that the health situation is worse in Myanmar than in neighboring countries. For example, in 2009, the infant mortality rate (IMR) was reported to be 54 per 1,000 live births in Myanmar, more than four times the rate in Thailand (12 per 1,000 live births) [1]. However, national figures do not include data from substantial portions of the country, including areas that are conflict-affected or controlled by non-state actors along Myanmar’s borders with Thailand and China.

The health information gap prior to the elections was partly due to the under-developed health research capacity in the country as a whole: Myanmar recently ranked 218^th^ out of 224 countries in number of publications in medicine per capita (0.4 per 100,000 people) [2]. Prior to 2010, over four decades of conflict had produced widespread internal displacement, erosion of health systems, and limited access to health care and information. Over 446,000 internally displaced people were living in heavily militarized areas of eastern Myanmar [3] where access for international humanitarian workers was severely constrained [4].

Community based organizations (CBOs) with unique access to these areas have filled health information gaps by conducting population-based surveys that have estimated rates of death and disability higher than rates reported for the country as a whole. For example, in 2004, CBOs conducted retrospective mortality surveys in Kayin (Karen) and Kayah (Karenni) [5] that found under-5 mortality rates of 218 per 1,000 live births, higher than the nationally reported rate of 105 per 1000 live births [6].

Rural poverty and a paucity of health care services contributed to poor health outcomes prior to elections in 2010 [7]. However, evidence suggests that economic and healthcare factors alone did not explain the elevated risk of disease and mortality in Myanmar’s border regions, where exposure to human rights violations (HRVs) also contributed to poor health. For example, loss of food security has been associated with anemia (hemoglobin < =11.0; odds ratio (OR) = 7.47) [8] and child malnutrition (OR = 1.94) [5]. Forced displacement has been also associated with unmet need for contraception (OR = 1.68) [8], child death (OR = 2.80), and child malnutrition (OR = 3.22) [5]. In Karen State, households that experienced assault during 2011 had 9 times the odds of moderate to severe household hunger. Forced labor was also associated with increased odds of night blindness (OR = 1.53) and diarrhea (OR = 2.98) [9].

Although widespread HRVs likely contributed to poor health outcomes in eastern Myanmar in 2004, evidence is lacking to illuminate the health and human rights conditions immediately prior to the 2010 elections. Since 2010, new cease-fire agreements have been signed with several non-state actors and qualitative reports suggest a relative decline in fighting in eastern Myanmar [10]. In order to provide a pre-election baseline from which the expected positive health-related benefits of political and economic change can be measured, this manuscript presents results of a large population-based survey of health and human rights conducted in eastern Myanmar, shortly before the recent transition. These data aim to estimate the burden of mortality and morbidity, the prevalence of HRVs, and the association between HRVs and poor health.

The present survey differs from previous studies in the region in several ways. As it was conducted via a joint effort of several CBOs working in partnership, the geographical scope and target population size are considerably larger. In addition, this survey was conducted across a variety of political contexts throughout eastern Myanmar, ranging from areas of ceasefire to ongoing conflict and fluctuating control.

## Methods

### Design

From October 2008-January 2009, 45 surveyors from participating CBOs conducted retrospective households surveys in four states and two divisions of eastern Myanmar, including accessible areas of Shan, Kayin, Bago, Kayah, Mon and Tanintharyi. The study was designed to estimate mortality rates within CBO service areas in eastern Myanmar. The sampling frame of 325,094 people and 57,950 households was constructed using population estimates collected by CBOs within approximately six months of survey implementation in their target service areas.

CBO partners surveyed internally displaced persons (IDP) living in designated camps where they provide services, but data from IDP camps were excluded from analyses presented in this manuscript because many camp residents have lived in camps for years, and their experience in the year prior to the survey does not reflect the health and human rights exposures of non-camp residents.

Three-stage cluster sampling was employed. The geographic boundaries of sampling strata were defined by the target service areas of the eleven participating CBOs. When organizations’ target populations were smaller than the required sample size needed to estimate stratum-specific mortality rates (n = 1,650 households in 55 clusters; see below for details), geographically contiguous organizations’ populations were pooled. The target populations of four proximal CBOs in Shan State and three proximal CBOs in Kayah State were combined; thus six strata comprised the first sampling stage. In the second stage, clusters were chosen within each stratum. During the third stage, proximity sampling was used to select 30 households in each cluster. A total of 8,130 surveys were planned in 274 village clusters selected proportionate to population size. Following exclusion of 21 clusters in IDP camps, the final intended sample for the results presented here included 7,500 households located in 253 villages. Households were defined as a group of people sharing meals under one roof. Surveyors were instructed to interview the woman in the household with the youngest child. If she was not available, the woman with the next oldest child was interviewed. If no woman was available, the male head of household was interviewed. Respondents were chosen in this way because many of the survey questions focus upon the health of mothers and children.

### Implementation

Surveyors received a two-week training in interviewing technique, sampling methods, case definitions, informed consent protocols, mid-upper arm circumference (MUAC) measurement, and rapid diagnostic tests for *Plasmodium falciparum* (Pf) malaria.

Surveys were translated into five local languages prior to field implementation, including Mon, Shan, Lahu, Karen (Po), and Burmese, and were administered by a native speaker of the respondents’ native language. Respondents were asked to enumerate the age and sex of all living household members and of those who had died in the previous year. Miscarriages, abortions, and stillbirths were not counted, as this study focused on calculation of mortality indicators using live births as a denominator. Respondents were asked to provide a perceived cause for each death. Complete reproductive histories were collected from female respondents. The majority of questions were asked with a one-year recall period with the exception of diarrhea (two weeks prior to the survey), oral rehydration solution (ORS) consumption (two weeks prior to the survey), vitamin A supplementation (six months prior to the survey), and landmine injuries (15 years prior to the survey). Acute malnutrition was assessed by measuring mid-upper arm circumference (MUAC) of women of reproductive age (15–49 years) and children 6–59 months of age. Infection with *Plasmodium falciparum* was tested using Paracheck rapid diagnostic tests (Paracheck-Pf® Orchid Biomedical Systems, Goa, India). Surveyors were instructed to conduct Paracheck tests on the respondent. A module on HRVs was included which queried each respondent about HRVs suffered by any member of their household during the past year; the rationale for the questions used in this module has been described elsewhere [5].

### Analysis

MUAC scores were compared to the World Health Organization Child Growth Standard MUAC values in centimeters for children age 6 to 59 months by sex and one-month age intervals [11]. Z-scores were calculated using the WHO package for Stata. Children who were less than 3 standard deviations (Z-score < -3) below the mean were classified as severely acutely malnourished; those with Z-scores below -2 were moderately acutely malnourished, and those below -1 were mildly acutely malnourished; others with Z-scores equal to or greater than -1 were classified as normal. Women with MUAC <22.5 cm were classified as malnourished [12].

IMR and under five mortality rate (U5MR) were estimated as a ratio of deaths to live births, while the crude mortality rate (CMR) and age-specific death rates were estimated as a ratio of all deaths to mid-year population. Prevalence was estimated for morbidity outcomes. For counts of deaths within households, rate ratios were estimated using Poisson regression with an offset for the number of household members at-risk for outcome events within relevant age groups. For individual-level binary outcomes, prevalence ratios were estimated using generalized linear models with a log link function.

The analysis was weighted with weights equal to the product of design and post-stratification weights. Design weights were equal to the inverse probability of being sampled within a stratum. Because the initial sampling frame included IDP camps, post-stratification weights were equal to the inverse probability of being in a non-camp household in a stratum. Data analysis was conducted using Stata 12.0, employing the *svy*: suite of commands when appropriate. Standard errors accounted for clustering at the village level.

In bivariate analyses, households that did not experience the specific abuse under scrutiny were included in estimates of measures of association. However, these households may have been exposed to other human rights abuses that increase their risk of adverse health outcomes. In order to assess the health impacts of exposure to HRVs compared to no exposure, an ordinal variable was created representing the cumulative exposure to HRVs (minimum = 0, maximum truncated at 5 HRVs).

### Sample size

The initial sample size was calculated for a cluster-sampled design to estimate under-five mortality rate (U5MR) for each stratum with a precision of +/- 60 deaths per 1,000 live births, assuming U5MR = 150/1,000, crude birth rate = 45/1,000, average household size = 5.6, and design effect = 2.5. This design effect was chosen based on previous health surveys in the region [5,13]. This calculation generated a required sample size of 1,350 households in 45 villages in each of six strata, assuming that 30 households would be interviewed per village cluster. We planned to collect data in 55 villages in case some villages could not be reached due to poor security. Following the exclusion of IDP camp populations as noted above, pooled data from the six survey strata permitted estimation of U5MR for the entire target population with a precision of approximately +/- 30 deaths per 1,000 live births.

### Ethics approval

Respondents provided oral consent to participate in the survey. Leadership of the Burma Medical Association, a civil society organization independent of the Myanmar government, provided primary ethical review of the study and survey itself. The Johns Hopkins Bloomberg School of Public Health Institutional Review Board approved secondary analysis of the data.

## Results

**Figure 1:**
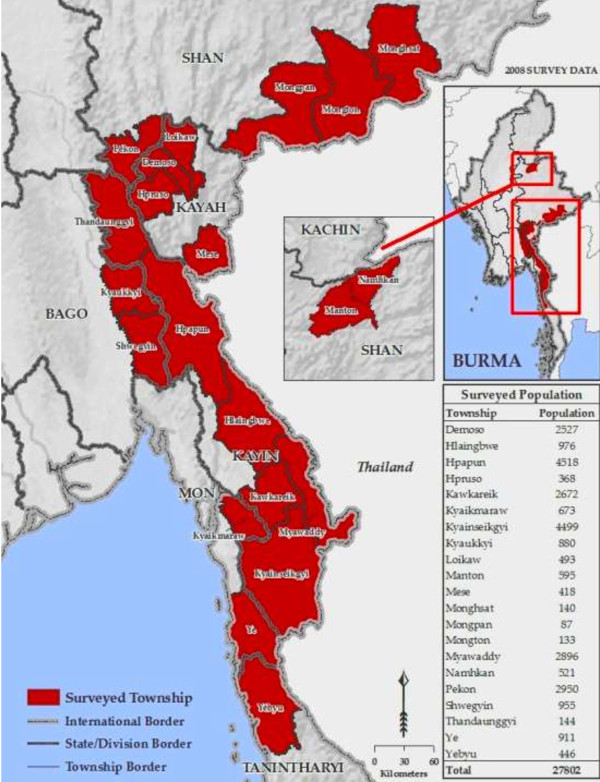
Surveyed areas.

A total of 5,748 households were reached out of the 7,500 surveys planned. Surveys were conducted in four states and two divisions; the majority were conducted in Kayin state (n = 3,363) and the fewest in Tanintharyi Division (n = 90). Figure 1 depicts the townships containing villages that were surveyed. As previously noted, the final number analyzed excludes surveys conducted in camps for IDPs. Surveyors were unable to reach all 253 villages as planned due primarily to security issues and ongoing conflict. Though surveyors were asked to substitute the nearest safe village (cluster) if they were unable to reach a pre-selected cluster, often a safe village was not available in the immediate geographic area due to the widespread nature of active conflict in the assigned target region. Thus of the 253 planned clusters, a total of 200 (79%) were reached. Similarly, in several regions surveyors were unable to complete 30 surveys before security concerns forced them to exit the village.

Among the 5,748 households that were invited to participate in the survey, 97.3% (n = 5,592) agreed to participate. The response rate was similar across all areas. The survey sample characteristics are summarized in Table 1.

**Table 1:** Target population, number of clusters and households surveyed, and basic demographic indicators

Sample** characteristic	**Indicator**
Target population	325,094
Number of clusters sampled	253*
Number of clusters reached	200*
Total households planned	7,500*
Total households reached	5,748*
Total consenting households	5,592*
Response rate	97.3%*
Mean household size	5.1**
Population <5 years old (%)	16.9**
Population <15 years old (%)	42.5**
Population >65 years old (%)	2.3**
Male to female ratio (all ages)	0.980**
Male to female ratio (15–25 years)	0.896**
Male to female ratio (15–45 years)	0.929**

*The initial protocol planned to survey 8,130 households; 7,500 households remained in the sampling frame following exclusion of 630 households located in in 21 IDP camp clusters. Results reported in this manuscript are limited to data from non-camp populations. See text for additional information.

**Results weighted using design and post-stratification weights.

### Demographics

Mean household size in this sample was 5.1. Overall, the ratio of males to females was 0.980. There was a deficit of males noted in the 15–25 year age range, with 90 males for every 100 females. This is commonly seen in areas of longstanding conflict, as young males are lost to war or migrate to seek employment opportunities outside of economically depressed regions.

Seventeen percent of the surveyed population were under age five, 42.5% under age 15, and 2.3% were over age 65, which reflects the relatively higher birthrate and mortality rate in eastern Myanmar compared to other nations in the region and mainland Myanmar itself.

### Mortality rates and morbidity

As shown in Table 2, 904 live births and 360 deaths were reported to have occurred in the year prior to the survey. Infant deaths accounted for a total of 68 of the deaths in this sample (Infant Mortality Rate (IMR) = 77; 95% CI 56 to 98), and there were 132 child deaths (U5MR = 139; 95% CI 107 to 171). The under-5 age-specific death rate (ASDR-5) was 28 (95% CI 22 to 34), and the crude mortality rate (CMR) was 13 (95% CI 11 to 15). The leading respondent-reported cause of death among all ages was malaria, which was reported to have caused 21.2% (95% CI 16.5 to 25.8) of deaths, followed by acute respiratory tract infections (ARI) and diarrhea, which were reported to cause 16.6% (95% CI 11.8 to 21.4) and 12.3% (95% CI 8.7 to 15.8) of deaths, respectively. Among children under five, neonatal causes were reported to be the leading cause of death (26.2%; 95% CI 17.6 to 34.9) followed by malaria (22.9%; 95% CI 14.7 to 31.1) and ARI (13.6%; 95% CI 5.5 to 21.8). Eleven deaths due to violent causes were reported (5 landmine fatalities and 6 gunshot fatalities) [Table 3].

**Table 2:** Mid-year population estimates, vital events, mortality rate estimates

	**Estimate**	**95% CI**
Mid-year population		
<5 years	4544	–
> = 5 years	22990	–
Vital events		
Live births	904	–
Infant deaths	68	–
Under 5 deaths	132	–
Overall deaths	360	–
Mortality rates/ratios		
Infant (IMR)	77	56 to 98
Under 5 (U5MR)	139	107 to 171
Age Specific Death 5 (ASDR-5)	28	22 to 34
Maternal Mortality (MMR)	711	150 to 1272
Overall (CMR)	13	11 to 15

**Table 3:** Reported causes of death

	**Count**	**Total**	Percent*	95% CI*
All ages				
Malaria	80	360	21.2	(16.5, 25.8)
ARI	61	360	16.6	(11.8, 21.4)
Diarrhea	48	360	12.3	(8.7, 15.8)
Other	78	360	22.2	(15.7, 28.7)
Neonatal	29	360	9.8	(6.3, 13.4)
Pregnancy-related	7	360	1.9	(0.5, 3.4)
Gunshot	6	360	2.1	(-0.2, 4.4)
Landmine	5	360	1.6	(-0.9, 4.2)
Don't know	46	360	12.2	(8.6, 15.8)
Under 5				
Malaria	33	132	22.9	(14.7, 31.1)
Diarrhea	21	132	12.5	(6.3, 18.7)
ARI	15	132	13.6	(5.5, 21.8)
Neonatal	29	132	26.2	(17.6, 34.9)
Other	15	132	12.7	(1.9, 23.5)
Don't know	19	132	12.1	(6.5, 17.6)

*Results were weighted with design and post-stratification weights.

Table 4 summarizes the prevalence of selected morbidities. Rapid diagnostic testing for *Plasmodium falciparum* malaria was performed on 5,074 (90.7%) respondents, 95% of whom were female; 418 of those tested (6.9%; 95% CI 5.7 to 8.1) were positive. MUAC, age, and sex data were collected for 3,234 children [Table 4] and 5,430 women of reproductive age [Table 5]. Mild acute malnutrition was found among 873 (27.2%; 95% CI 24.4 to 30.0) children, moderate among 309 (9.6%; 95% CI 7.4 to 11.7) children, and severe among 156 (5.2%; 95% CI 3.2 to 7.2) children. Respondents reported diarrhea among 10.3% of children under five during the past two weeks (95% CI 8.4 to 12.1).

**Table 4:** Prevalence of Selected Morbidities

**Outcomes**	**N**	n*	% (95% CI)*
Plasmodium falciparum (Pf) positive (heads of household)	5074	418	6.9 (5.7 to 8.1)
Acute child malnutrition			
Mild	3234	873	27.2 (24.4 to 30.0)
Moderate	3234	309	9.6 (7.4 to 11.7)
Severe	3234	156	5.2 (3.2 to 7.2)
Moderate/severe	3234	465	14.8 (11.5 to 18.1)
Child diarrhea in previous 2 weeks	4329	471	10.3 (8.4 to 12.1)

*Results were weighted with design and post-stratification weights.

**Table 5:** Use of iron supplementation and malnutrition among women of reproductive age

**Outcomes**	**Shan**		**Bago**		**Kayin**		**Mon**		**Kayah**		**Tanintharyi**		**All areas**	
	**n (%)**	**95% CI**	**n (%)**	**95% CI**	**n (%)**	**95% CI**	**n (%)**	**95% CI**	**n (%)**	**95% CI**	**n (%)**	**95% CI**	**n (%)**	**95% CI**
Use of Iron Supplementation, any	278 (31.8)	(24.8, 38.8)	163 (50.1)	(42.0, 58.2)	1360 (44.0)	(39.0, 48.9)	70 (36.5)	(19.0, 54.1)	201 (37.5)	(28.5, 46.5)	44 (69.8)	(57.6, 82.0)	2116 (42.2)	(38.7, 45.7)
Use of Iron Supplementation, at least 90 days	26 (3.9)	(1.5, 6.3)	27 (8.2)	(4.0, 12.4)	827 (23.0)	(18.5, 27.5)	32 (15.1)	(-3.1, 33.4)	37 (6.6)	(1.9, 11.4)	19 (30.2)	(8.2, 52.2)	968 (17.1)	(14.3, 19.9)
Malnutrition, women of reproductive age (15-49 y.o.)*	118 (11.9)	(6.5, 17.3)	94 (28.5)	(19.0, 38.0)	468 (17.0)	(13.4, 20.7)	107 (29.1)	(15.1, 43.0)	40 (4.0)	(1.4, 6.6)	24 (28.9)	(18.4, 39.5)	851 (16.7)	(13.9, 19.5)

* MUAC data collected on a total of 5430 women.

Among women ages 15 to 49, 851 (16.7%; 95% CI 13.9 to 19.5) were malnourished (MUAC <22.5 cm) [Table 5]. Malnutrition among women ranged from a low of 4.0% (95% CI 1.4-6.6) in Kayah State to a high of nearly 30% in Bago, Mon, and Tanintharyi states. Maternal malnutrition has been found to increase risk of low birth weight and intrauterine growth restriction [14]. Additionally, the majority of female respondents did not report taking iron supplements during their last pregnancy: 42.2% (95% CI 38.7 to 45.7) reported any use of iron during their last pregnancy, while 17.1% (95% CI 14.3 to 19.9) reported use of iron supplementation for at least 90 days. Maternal anemia has been associated with an increased risk of pre-term labor, low birth weight, and higher infant mortality [15]. Anemia is known to be highly prevalent in eastern Myanmar, as high as 66.2% among surveyed women in 2008 [16].

### Human rights violations

All 5,592 respondents that consented to participate in this survey provided information about HRVs perpetrated against household members during the year preceding the survey. Over one third of households suffered at least one violation in the preceding year (36.2%; 95% CI 30.7 to 41.7). The most common HRVs seen in surveyed areas were destruction and seizure of food, livestock, or crops (16.1%%; 95% CI 12.0 to 20.1), forced cultivation of jatropha, an inedible biodiesel plant, (10.4%; 95% CI 7.1 to 13.7) and forced labor (13.3%; 95% CI 10.1 to 16.5). Respondents also reported forced displacement (5.5%; 95% CI 2.2 to 8.7), physical injuries, including gunshot wounds, landmine injuries, beatings, stabbings (5.0%; 95% CI 3.8 to 6.2), and being detained or tied up (2.5%; 95% CI 1.0 to 4.1) [Table 6].

**Table 6:** Human rights violations, overall and by state

**Violation**	Shan*	95% CI*	Bago*	95% CI*	Kayin*	95% CI*	Mon*	95% CI*	Kayah*	95% CI*	Tanintharyi*	95% CI*	**All areas**	95% CI*
Forced labor	267 (51.2%)	(42.2, 60.2)	30 (9.8%)	(-8.6, 28.2)	365 (10.1%)	(5.8, 14.4)	0 (0.0%)	n/a	1 (0.1%)	(-0.1, 0.4)	2 (2.2%)	(-1.4, 5.9)	665 (13.3%)	(10.1, 16.5)
Destruction and seizure of food, livestock, or crops	248 (56.4%)	(48.1, 64.6)	67 (22.1%)	(1.7, 42.5)	244 (9.7%)	(4.3, 15.1)	53 (18.1%)	(-3.6, 39.8)	5 (0.7%)	(0.0 to 1.4)	4 (4.4%)	(-2.8, 11.7)	621 (16.1%)	(12.0, 20.1)
Forced to grow jatropha	37 (6.8%)	(-4.5, 18.2)	0 (0.0%)	n/a	74 (1.4%)	(0.4, 2.5)	179 (38.3%)	(13.3, 63.4)	392 (56.8%)	(39.1, 74.4)	0 (0.0%)	n/a	682 (10.4%)	(7.1, 13.7)
Displacement	12 (2.7%)	(-1.8, 7.2)	66 (21.2%)	(-0.8 to 43.2)	148 (5.5%)	(1.5, 9.5)	0 (0.0%)	n/a	3 (0.5%)	(0.0 to 0.9)	0 (0.0%)	n/a	229 (5.5%)	(2.2, 8.7)
Physical injuries (gunshot wounds, landmine injuries, beatings, stabbings)	70 (15.8%)	(12.0, 19.6)	6 (1.8%)	(0.5 to 3.1)	100 (3.4%)	(2.2, 4.7)	26 (8.9%)	(-3.4, 21.2)	15 (2.2%)	(1.3, 3.1)	0 (0.0%)	n/a	217 (5.0%)	(3.8, 6.2)
Detained or tied up	37 (8.4%)	(3.1, 13.8)	0 (0.0%)	n/a	21 (0.8%)	(0.1, 1.4)	40 (13.6%)	(-4.4, 31.7)	5 (0.7%)	(0.0, 1.4)	0 (0.0%)	n/a	103 (2.5%)	(1.0, 4.1)
Number of HRVs experienced by household														
0	526 (35.6%)	(27.2, 43.9)	213 (62.2%)	(38.5, 86.1)	2473 (75.0%)	(68.3, 81.7)	150 (43.6%)	(16.5, 70.6)	292 (42.4%)	(25.3, 59.6)	85 (94.4%)	(85.4, 103.5)	3739 (63.8%)	(58.3, 69.3)
1	107 (12.9%)	(7.8, 18.0)	67 (21.4%)	(3.6, 39.3)	621 (20.8%)	(14.7,, 27.0)	182 (39.4%)	(14.7, 64.1)	378 (54.5%)	(38.5, 70.5)	4 (4.4%)	(-2.8, 11.7)	1359 (24.5%)	(19.6 to 29.4)
2	78 (17.6%)	(11.3, 23.9)	22 (7.1%)	(-1.0 to 15.3)	96 (2.5%)	(1.2 to 3.8)	5 (1.7%)	(-0.5, 3.9)	19 (2.8%)	(1.0, 4.6)	0 (0.0%)	n/a	220 (4.7%)	(3.4, 6.0)
3	78 (17.8%)	(12.6, 22.9)	28 (9.1%)	(-4.9, 23.2)	29 (0.9%)	(0.3 to 1.6)	6 (2.0%)	(-0.7, 4.8)	2 (0.3%)	(0.0 to 0.6)	1 (1.1%)	(-0.7 to 2.9)	144 (3.7%)	(2.3, 5.1)
4	43 (9.8%)	(6.0, 13.6)	0 (0.0%)	n/a	10 (0.4%)	(0.1 to 0.6)	16 (5.5%)	(-1.3, 12.2)	0 (0.0%)	n/a	0 (0.0%)	n/a	69 (1.8%)	(1.1, 2.5)
5 or more	28 (6.4%)	(2.8 to 10.0)	0 (0.0%)	n/a	10 (0.4%)	(-0.1 to 0.8)	23 (7.8%)	(-3.6, 19.3)	0 (0.0%)	n/a	0 (0.0%)	n/a	61 (1.5%)	(0.5, 2.5)

*Percentages and standard errors were weighted with design and post-stratification weights.

The percent of households experiencing each type of violation varied greatly across states [Table 6]. A higher percentage of households in Shan, Mon, and Kayah states reported HRVs in the previous year than in other states. In Shan State, over half of respondents reported being forced to do labor (51.2%; 95% CI 42.2 to 60.2) or having food, livestock, or crops destroyed or seized (56.4%; 95% CI 48.1 to 64.6). In Bago State, 22.1% (95% CI 1.7 to 42.5) of households reported destruction and seizure of food, livestock or crops. Participants in Mon and Shan States were more likely to report physical injuries and detainment than participants in other areas.

Patterns of forced labor varied considerably across regions. Among households in Mon and Kayah states, where ceasefire agreements have been relatively more successful at preventing violence, forced growing of jatropha was common (38.3%; 95% CI 13.3 to 63.4 and 56.8%; 95% CI 39.1 to 74.4, respectively). In contrast, in Shan, Bago and Kayin states, which have a more recent history of conflict, less than 10% of households were forced to grow jatropha, and forced labor was more common. In surveyed areas of Bago and Kayin, 9.8% (95% CI -8.6 to 28.2) and 10.1% (95% CI 5.8 to 14.4) of households, respectively, were forced to work without compensation.

### Associations between human rights violations and health outcomes

The prevalence and rates of selected health outcomes were compared between households experiencing three categories of HRVs and households not experiencing those specific violations. Potential confounding of the association between exposure and outcome variables by household size was explored; in adjusted models, household size was not statistically significant, and adjustment did not substantially alter measures of association. As such, unadjusted ratios pooled across all survey areas are presented in Table 7.

**Table 7:** Morbidity and mortality risk by exposure to HRVs

	**Exposed**			**Non-exposed**			**Rate/ prevalence ratio**^**a**^	**95% CI**
	**Total**	**n**	**%**^**a**^	**Total**	**n**	**%**^**a**^		
**Forced labor**								
Infant mortality	665	19	3.4	4899	48	1.0	2.2^b^	1.1 to 4.4
Under 5 mortality	665	28	5.0	4899	103	2.0	2.1^b^	1.3 to 3.6
Crude mortality	665	56	9.4	4899	301	5.8	1.5^b^	1.1 to 2.1
Child diarrhea (2 weeks)	625	66	9.3	3680	401	10.4	0.9	0.6 to 1.4
Pf positive	583	42	7.0	4478	376	7.0	1.0	0.6 to 1.7
Moderate-severe acute malnutrition	444	19	5.1	2772	445	16.5	0.3^b^	0.2 to 0.6
**Displacement**								
Infant mortality	229	5	2.1	5336	62	1.3	1.4	0.6 to 3.4
Under 5 mortality	229	10	4.2	5336	121	2.3	1.4	0.7 to 3.0
Crude mortality	229	18	7.7	5336	339	6.2	1.1	0.6 to 2.0
Child diarrhea (2 weeks)	238	34	14.5	4067	431	9.8	1.5	0.9 to 2.5
Pf positive	210	10	4.8	4852	406	7.0	0.7	0.3 to 1.3
Moderate-severe acute malnutrition	192	79	41.6	3025	385	12.7	3.3 ^b^	1.9 to 5.6
**Destruction or seizure of food, livestock, or crops or being forced to give food**								
Infant mortality	621	15	2.6	4825	52	1.1	1.7	1.0 to 3.1
Under 5 mortality	621	23	3.9	4825	108	2.2	1.5	0.9 to 2.3
Crude mortality	621	47	7.8	4825	309	6.2	1.2	0.9 to 1.7
Child diarrhea (2 weeks)	585	59	9.6	3609	395	10.3	0.9	0.7 to 1.3
Pf positive	555	44	8.3	4414	370	6.7	1.2	0.8 to 2.0
Moderate-severe acute malnutrition	334	61	17.8	2798	396	14.4	1.2	0.7 to 2.1

^a^Results were weighted with design and post-stratification weights.

^b^p < 0.05.

The infant, under-5 and crude mortality rates in households that had experienced forced labor were significantly higher than in households that did not experience forced labor (rate ratio = 2.2; 95% CI 1.1 to 4.4; RR = 2.1; 95% CI 1.3 to 3.6; RR = 1.5; 95% CI 1.1 to 2.1). The prevalence of moderate to severe acute malnutrition among children 6–59 months was three times higher among households that were displaced (prevalence ratio = 3.3; 95% CI 1.9 to 5.6). Similarly, the prevalence of diarrhea in the prior two weeks was increased among children in households that were displaced compared to those in households that were not displaced, though the association was not statistically significant (PR = 1.5; 95% CI 0.9 to 2.5). The rate of under-5 (RR = 1.5; 95% CI 0.9 to 2.3) and infant mortality (RR = 1.7; 95% CI 1.0 to 3.1) was higher in households who experienced destruction or seizure of food, livestock, or crops or were forced to give food, though these findings did not reach statistical significance. Forced cultivation of jatropha was not associated with health or mortality outcomes in bivariate analyses.

As shown in Table 8, the under-5 age-specific death rate (ASDR-5) and CMR were higher among households that experienced two or more violations (RR = 2.0; 95% CI 1.3 to 3.2 and RR = 1.5; 95% CI 1.1 to 2.1, respectively). The prevalence of diarrhea among children under five was higher among households experiencing two or more HRVs, but the association was not statistically significant. The prevalence of moderate to severe malnutrition was higher among families that experienced one HRV (RR =1.4; 95% CI 1.0 to 2.1), though this relationship did not reach statistical significance.

**Table 8:** Morbidity and mortality risk by cumulative exposure to human rights violations

**Outcome**	**Deaths**	**Mid-year population/respondents**	Ratio * 1000^**a**^	**Rate ratio**^**a**^	**95% CI**^**a**^
ASDR-5					
No violations reported	83	2968	24.2	1.0	– to –
One violation	26	1064	25.4	1.0	0.6 to 2.0
Two or more violations	23	512	49.3	2.0	1.3 to 3.2
CMR					
No violations reported	239	18108	12.3	1.0	– to –
One violation	73	6709	11.5	0.9	0.7 to 1.3
Two or more violations	48	2718	18.8	1.5	1.1 to 2.1
	**Cases**	**Respondents**	**Prevalence**^**a**^	**Prevalence ratio**^**a**^	**95% CI**^**a**^
Pf positive					
No violations reported	308	3410	7.1	1.0	– to –
One violation	75	1226	6.0	0.8	0.6 to 1.3
Two or more violations	35	438	8.1	1.1	0.7 to 2.0
Under-5 diarrhea					
No violations reported	309	2839	10.2	1.0	– to –
One violation	104	1002	10.1	1.0	0.7 to 1.4
Two or more violations	58	488	10.9	1.1	0.7 to 1.6
Moderate-Severe Malnutrition					
No violations reported	277	2139	13.1	1.0	– to –
One violation	143	815	18.6	1.4	1.0 to 2.1
Two or more violations	45	280	17.0	1.3	0.6 to 2.7

^a^Results were weighted with design and post-stratification weights.

## Discussion

This large survey demonstrates that prior to elections in 2010, populations in eastern Myanmar experienced a high risk of death, disease and human rights violations. The survey represents the largest coordinated research effort to date of multiple civil society organizations in eastern Myanmar to document the health, mortality, and human rights situation, and it complements and extends the findings from previous studies in smaller, targeted areas.

### Morbidity and mortality

The estimated prevalence of *Plasmodium falciparum* malaria (6.9%) and under-5 diarrhea (10.3% in the previous 2 weeks) were comparable to a previous survey in Karen State (11.2% and 13.5%, respectively), though the estimate of moderate and severe malnutrition (14.8%) was higher (previously 4.2%) [5,8]. Maternal health indicators were poor, and were similar to those reported from selected clinic service areas participating in a reproductive health intervention study in 2008: only 17.1% of women of reproductive age met the international recommendation for consumption of iron supplements during pregnancy (previously 11.8%) [8], and nearly one fifth of women of reproductive age were found to be malnourished (16.7% in this study, 19.3% previously) [8].

Although the point estimates for mortality rates in this survey were lower than those previously recorded in this region [5], confidence intervals overlapped. Under-5 and infant mortality rates for eastern Myanmar were higher than contemporary national rates for Myanmar reported by UNICEF (139 versus 71 and 77 versus 54, respectively) [1]. The standard errors of national parameter estimates were not available to calculate the 95% confidence interval around the UNICEF point estimates. However, the lower bound of the 95% confidence interval around the estimated under-five mortality rate in the present study (107) was substantially higher than 71, making it highly likely that U5MR in the study area is higher than the national average. The finding that the majority of deaths in this sample are reported to be due to preventable causes confirms previous findings in the region [5].

### Human rights violations

A wide and growing body of literature on the human rights situation in eastern Myanmar exists. In most cases, findings of this survey corroborate findings published in the qualitative literature for the period of time covered by the survey [3,17,18].

The finding that over one third of households experienced at least one type of human rights violation in this survey and that the percentage of households experiencing at least one violation ranged from 12.9% to 54.5% across multiple states (exclusive of Tanintharyi, for which the sample was relatively small) suggest that HRVs were widespread throughout eastern Myanmar prior to the recent political transition. Furthermore, HRVs were associated with child malnutrition and an increased risk of death among infants, children and the population as a whole. Forced labor was most strongly associated with infant and child death; it was associated with a *decreased* risk of child malnutrition, a finding which has been reported elsewhere, though the reasons for this finding remain unclear and require further study [5]. However, many households that did not experience forced labor were exposed to other HRVs that also increase risk for malnutrition, such as forced displacement, thus making it possible that the "control" group in this instance had greater exposure to alternative, stronger risk factors for child malnutrition. The positive association observed in this study between displacement and malnutrition has been noted in qualitative studies of Karen State [17]. This survey did not confirm the previously documented association between forced displacement and prevalent *Plasmodium falciparum*[5].

### Limitations

Although this study’s cross-sectional design does not allow for causal inference, there are potential feasible causal pathways between HRVs and health that have been described elsewhere [5]. Respondents' recall limitations could have led to bias, particularly for mortality outcomes. Because mortality cases and causes of death were reported by surviving household members without verbal autopsy or physician-classified cause of death, it is possible that poor knowledge of health and lack of access to health facilities and practitioners resulted in misclassification of causes of death. Surveyors were trained to ask probing follow-up questions to determine the cause of death as precisely as possible. Surveys were translated and administered in five local languages to attempt to minimize information bias, and surveyors were trained to use locally familiar dates as time anchors to assist respondents with time intervals and minimize recall bias.

The HRVs module did not include questions about the timing of events, the perpetrator, or the duration and frequency of exposures. Each of these factors could bias estimated associations between HRVs and health outcomes. It is possible that respondents censored their responses out of fear. Conversely, it is possible that respondents exaggerated the violations they experienced with the hopes of drawing more attention and assistance to these issues in their communities.

Since the geographic area of the study was based on service provision by participating organizations, it is possible that the sampling frame excluded areas where security is too poor for service implementation. Although mobile teams of participating organizations deliver services in highly insecure areas, it was not possible to collect and return data from several particularly dangerous areas. It is possible, if not likely, that the health and human rights situations in such areas are worse than in those for which data was available. Considering these limitations, the returned results likely present a "best-case" scenario.

Surveys were administered at the end of the rainy season, and this may have resulted in lower malaria and diarrhea morbidity, which are likely to be higher during the rainy season. Prevalence of malaria among predominantly female heads of household is lower than general populations in eastern Myanmar, and by testing only interviewees, the present protocol likely underestimates the burden of malaria in the population as a whole [19]. Care should be taken in comparing such rates to those from other surveys conducted during other seasons.

Point estimates of morbidity and mortality indicators represent an average across a diverse range of populations in eastern Myanmar and may not reflect the burden of disease or death within a specific area. Additionally, several estimates lacked precision due to constraints of time and sampling limitations, and these wider confidence intervals must be carefully accounted for when interpreting findings and comparing estimates to regional and national figures.

Prevalence of HRVs varied dramatically by state, and associations between mortality and morbidity outcomes were estimated based on data pooled across administrative regions. Thus, these associations may be confounded by factors related to the diversity of the surveyed populations. However, this bias might also lead to an underestimation of true associations. Causal pathways for the impact of HRVs on health have been discussed by other authors [5], and similar associations in Myanmar have been described by other authors in multiple studies [5-9], contributing to the plausibility of these findings.

## Conclusion

These findings provide evidence for high rates of HRVs and poorer health status in eastern Myanmar, including regions affected by active conflict prior to the recent national transition. These data add to a growing body of literature demonstrating the association between health and human rights. Public health interventions may be less likely to succeed if they are not complemented by concerted efforts to eliminate HRVs. International and domestic actors may use these data as a baseline to measure the impact of recent political change and economic investment in eastern Myanmar on HRVs and health status.

## Competing interests

The authors declare they have no competing interests.

## Authors’ contributions

PKP contributed to data analysis and drafted the manuscript. JBC contributed to design of the survey and training of surveyors, led the management of data collection and data entry, conducted statistical analysis and contributed to manuscript drafting. LS contributed to design of the survey, training of surveyors, data analysis and critically revised the manuscript. SNH participated in its design and coordination and helped to review the manuscript. EKSO, DR, CM, MM, AL and SL participated in its design and coordination and helped to review the manuscript. TL contributed to design of the survey, data analysis, and critically revised the manuscript. AKR participated in the design of the study, designed and performed the statistical analysis with JBC, and critically revised the manuscript. All authors read and approved the final manuscript.

## Pre-publication history

The pre-publication history for this paper can be accessed here:

http://www.biomedcentral.com/1472-698X/14/15/prepub
